# An Unusual Etiology of Hypothyroidism and New-Onset Insulin-Dependent Diabetes: A Rare Side Effect of Nivolumab

**DOI:** 10.7759/cureus.24463

**Published:** 2022-04-25

**Authors:** Halah Alchalabi, Safa Albustani, Nusha Fareen, Ndausung Udongwo, Saira Chaughtai, Soemiwati Holland

**Affiliations:** 1 Internal Medicine, Jersey Shore University Medical Center, Neptune Township, USA; 2 Endocrinology, Diabetes and Metabolism, Jersey Shore University Medical Center, Neptune Township, USA

**Keywords:** checkpoint inhibitors, nivolumab induced insulin-dependent diabetes, nivolumab induced hypothyroidism, hypothyroidism, insulin-dependent diabetes, opdivo, nivolumab

## Abstract

Nivolumab, a monoclonal antibody targeting programmed cell death 1 receptor, is prescribed for many advanced cancers like malignant melanoma, non-small cell lung cancer, renal cell cancer, etc. With the increase in the use of nivolumab like immunotherapy, the incidences of immune-related side effects are also on the rise. Immune-related endocrinopathies like hypothyroidism and new-onset type 2 diabetes mellitus associated with nivolumab use, although rare, are already documented in the literature. Here we present a case of hypothyroidism and new-onset type 2 diabetes mellitus in a renal cell cancer patient receiving nivolumab for the past six months. The patient was managed successfully with discontinuation of nivolumab, intravenous insulin, and thyroid hormone replacement therapy. These types of endocrinopathies can be fatal; hence, prompt diagnosis and management are required. Thus, not only physicians’ awareness of such endocrinopathies among nivolumab-treated patients but also patients’ awareness regarding warning signs and symptoms are essential.

## Introduction

Immune checkpoint inhibitors have become one of the counter-stone treatment recommendations for solid tumors over the years [1]. Programmed death-1 receptor (PD-1) inhibitors have shown an excellent response in the management of several malignancies including but not limited to small-cell lung cancer, melanoma, and renal cell carcinoma (RCC) [2]. They work by inhibiting the receptor expressed on activated T-cells, thereby reversing immune suppression and releasing T-cell activation [2]. Nivolumab, also sold under the brand name OPDIVO® (Bristol Myers Squibb, New York, United States), is a human immunoglobulin G4 PD-1 immune checkpoint inhibitor antibody that inhibits PD-1 and promotes antitumor immunity [3]. While its use enhances cancer therapy, an increased risk for immune-related adverse effects like thyroid disease (hypo/hyperthyroidism), pancreatitis, and hypothyroidism have been reported in the literature [2-4]. Without prompt diagnosis and adequate management, such endocrinopathies could lead to fatal outcomes. Several published reports have documented nivolumab-induced thyroid dysfunctions [4-10]. A systematic review and meta-analysis published by Barroso-Sousa and his colleagues documented incidences of different endocrinopathies following the use of immune checkpoint inhibitors including nivolumab [4]. As per their study, the prevalence of hypothyroidism in patients receiving nivolumab can be as high as 6.5% and thyrotoxicosis (as evidenced by a low level of TSH) can present in 2.5% of the patients [4]. The same study also noted that compared to the incidence of thyroid dysfunction, the number of insulin-deficient diabetes mellitus cases following anti-PD-1 therapy, although less, is not unheard of [4].

Here we present a case of hypothyroidism and hyperosmolar hyperglycemic state in a 59-year-male with a history of right RCC status post nephrectomy and receiving nivolumab (started about six months prior). Presenting symptom was chest pain. On admission, clinical findings and laboratory parameters ruled out acute coronary syndrome and established the diagnosis of hypothyroidism with hyperosmolar hyperglycemic status in the background of new-onset type 2 diabetes mellitus. The patient was managed successfully with discontinuation of nivolumab, intravenous insulin therapy, correction of electrolyte imbalance, and thyroid hormone replacement therapy. To the best of our knowledge, there are only a few endocrinopathy cases reported following the administration of nivolumab.

## Case presentation

Our patient was a 59-year-male with a past medical history of RCC managed with nephrectomy. The patient also had a history of non-ischemic cardiomyopathy with an ejection fraction of 25-30% on goal-directed therapy (GDT)/implantable cardioverter-defibrillator, hypertension, hyperlipidemia, and chronic kidney disease stage 3. He reported to the emergency department with symptoms of chest pain for the past 16 hours. Symptoms occurred at rest, 5/10 in severity, and non-radiating. It was relieved by sublingual nitroglycerine en route to the hospital. It was associated with fatigue, nausea, polyuria, and polyphagia. He denied any fever, bowel changes, trauma, recent travel history, and any history of pre-diabetes/diabetes or thyroid disease. Of note, he was placed on nivolumab for his RCC about six months before this admission. His other home medications were metoprolol succinate, spironolactone, sacubitril-valsartan, furosemide, aspirin, and atorvastatin. His mother had a history of type 2 diabetes. He did not have any history of tobacco or illicit drug abuse in the past and only drank alcohol occasionally. 

On initial assessment, his vitals were: blood pressure, 121/83 mmHg; heart rate, 85 beats per minute; respiratory rate, 20 breaths per minute; temperature, 97.8℉; and oxygen saturation of 97% on room air. His oral mucosa appeared dry. The cardiovascular examination was unremarkable with no murmurs, rubs, gallops, or jugular venous distention. The pulmonary examination was also unremarkable. He had trace bilateral pedal edema. An electrocardiogram showed sinus rhythm with normal pulse rate and without any abnormality of ST-segment and T wave. Troponin level was 0.01 ng/mL (normal: <0.04 ng/mL) and chest x-ray findings were within normal limits. 

Initial labs showed significantly elevated levels of glucose and thyroid-stimulating hormone (TSH) levels as shown in Table 1. Urinalysis was positive for ketones; 20 mg/dL (normal: negative). Venous blood revealed a pH level of 7.344 as shown in Table 1. An acute coronary syndrome was ruled out, and he was admitted for management of hyperosmolar hyperglycemic state (HHS) and hypothyroidism. Due to a low level of C-peptide, <0.5 ng/mL (1.1-4.4 ng/mL), and a high level of TSH as shown in Table 1, a new-onset insulin-dependent autoimmune diabetes and hypothyroidism induced as a side effect of nivolumab was suspected. 

**Table 1:** Clinical Laboratory Parameters pH: potential hydrogen; PaCO2: partial pressure of carbon dioxide; paO2: partial pressure of oxygen.

Variable	Result	Reference range, adult
Hemoglobin (g/dL)	14	13.5-17.5
White cell count (x10^9^/L)	6.1	4.5-10.5
Sodium (mmol/L)	121	135-145
Serum osmolality (mOsm/kg)	333	280-300
Potassium (mmol/L)	5.5	3.5-5.2
Chloride (mmol/L)	92	98-107
Beta-hydroxybutyrate (mmol/L)	1.52	0.02-0.27
Anion gap (mmol/L)	15	5-13
Bicarbonate (mmol/L)	20	22-29
Glucose (mg/dL)	1090	70-100
Blood urea nitrogen (mg/dL)	30	8-24
Creatinine (mg/dL)	2.07	0.8-1.23
Bilirubin, total (mg/dL)	2.3	≤ 1.2
Calcium (mg/dL)	8.2	8.9-10.1
Alkaline phosphatase (U/L)	70	45-115
Alanine aminotransferase (U/L)	50	8-45
Aspartate aminotransferase (U/L)	35	8-45
Lipase (U/L)	118	7-60
B-type natriuretic peptide (ng/dL)	135	≤ 67
Troponin (ng/ml)	0.01	≤ 0.04
Thyroid-stimulating hormone (IU/L)	> 50,000	0.3-4.2
Hemoglobin A1C (%)	8.4	< 6.5
C-peptide (ng/ml)	< 0.5	1.1-4.4
Venous gas		
pH	7.344	7.35-7.45
PaCO2 (mmHg)	39.6	42-55
PaO2 (mmHg)	40	30-50

Nivolumab was discontinued from his medication regimen. Endocrinology was consulted for the management of HHS and hypothyroidism. Insulin drip and levothyroxine were started, and he was transferred to the intensive care unit for observation. Thereafter, his metabolic derangements improved with the resolution of hyperglycemia and electrolyte disturbances. On discharge, his symptoms improved and he was advised to stop taking nivolumab. He continued to follow up with the endocrinologist as an outpatient and has been compliant with his insulin therapy and levothyroxine for treatment of insulin-dependent diabetes and hypothyroidism, respectively.

## Discussion

A literature review reveals that there are several published case reports describing hypothyroidism occurring as a consequence of nivolumab therapy prescribed in different types of cancers. Kastrisiou and his colleagues reported a case of nivolumab-induced hypothyroidism and selective pituitary insufficiency in a patient with adenocarcinoma lung [5]. In their report, nivolumab therapy led to stable disease after seven cycles and the patient tolerated the drug quite well without any adverse events. However, after the 11th cycle, he complained of fatigue, anorexia, gait abnormality, and confusion; clinical findings were significant for bradycardia and pedal edema. Thyroid profile revealed abnormally high TSH with marked fall in T3 and T4 establishing the diagnosis of hypothyroidism. Although thyroid replacement therapy led to the improvement of biochemical parameters, his symptoms deteriorated. Clinical and finally laboratory findings established adrenocortical insufficiency. Following nivolumab therapy discontinuation, the patient was successfully managed with corticosteroid and thyroid replacement therapy.

In another article by Iadarola et al., two cases of nivolumab-induced thyroid dysfunctions are reported [6]. Also, Lin and his colleagues reported nivolumab-induced hypothyroidism following colon surgery in a 73-year-old female patient suffering from colorectal cancer. The patient became symptomatic following four cycles of nivolumab therapy. After the stoppage of nivolumab therapy and initiation of thyroid replacement therapy, her symptoms were relieved and laboratory parameters normalized.

Like our patient who developed new-onset diabetes mellitus presenting as the hyperosmolar hyperglycemic state (HHS) besides hypothyroidism, Saleh et al. presented a case of diabetic ketoacidosis along with HHS in a patient receiving nivolumab for metastatic testicular lymphoma [7]. The patient was managed successfully with intravenous fluid administration, electrolyte replacement, and insulin infusion. The patient was discharged once the laboratory parameters were normalized with multiple daily doses of insulin and nivolumab was started again.

In another report by Yilmaz, a case of nivolumab-induced type 1 diabetes mellitus was documented in a 49-year-old male patient suffering from stage IV renal cell carcinoma [8]. Following initiation of nivolumab therapy, the patient complained of increased sleepiness, loss of body weight, increased thirst, and increased frequency of urination. The patient was diagnosed with diabetic ketoacidosis and with low C peptide indicating diabetic ketoacidosis in the background of new-onset diabetes mellitus.

In our case report, the 59-year-old patient with RCC, receiving nivolumab for the past six months, presented to the emergency department with chest pain. On admission, the clinical and laboratory findings established the diagnosis of hypothyroidism along with HHS. Before starting nivolumab therapy, the patient’s thyroid profile and blood glucose levels were within normal limits. After stopping nivolumab, the patient was managed satisfactorily with intravenous insulin therapy, electrolyte imbalance correction, and thyroid replacement therapy.

On follow-up after two weeks, the patient was doing satisfactorily clinically and in his laboratory parameters with the stopping of nivolumab, insulin replacement, and levothyroxine. Despite the discontinuation of nivolumab, the patient had stable malignancy at the time of follow-up after three months.

Immune-related endocrinopathies following immunotherapy are already well-documented side effects. Commonly reported immune-related side effects are thyroiditis, hypophysitis, adrenalitis, and diabetes mellitus [4]. In a systematic review and meta-analysis published by Barroso-Sousa et al., diabetes mellitus was documented in about 0.2% of patients out of the 7551 subjects [4]. It was also reported that the majority of the patients with checkpoint inhibitor-induced diabetes mellitus presented with diabetic ketoacidosis [8-10]. The already documented cases also report that none of the patients had any glycemic abnormality or thyroid dysfunction. The literature also shows that the presence of certain human leukocyte antigens (HLAs) subtypes like DRB1*03 or 04 indicated more risk of developing checkpoint inhibitor-related diabetes mellitus [8-10]. The history of our patient revealed that his mother had a history of type 2 diabetes mellitus.

## Conclusions

With the wide use of checkpoint inhibitors, incidences of immune-related endocrinopathies such as hypothyroidism and diabetes mellitus are on the rise. Delay in diagnosis and management can be life-threatening in such cases. Hence, physicians must remain vigilant about endocrinopathies among patients receiving immunotherapy like nivolumab therapy and also educate patients and their caregivers about potential warning signs and symptoms. Additionally, periodic laboratory monitoring for endocrinopathies is also warranted to ensure early diagnosis and prompt management.
